# Novel PET/SPECT Probes for Imaging of Tau in Alzheimer's Disease

**DOI:** 10.1155/2015/124192

**Published:** 2015-03-23

**Authors:** Hiroyuki Watanabe, Masahiro Ono, Hideo Saji

**Affiliations:** Department of Patho-Functional Bioanalysis, Graduate School of Pharmaceutical Sciences, Kyoto University, 46-29 Yoshida Shimoadachi-cho, Sakyo-ku, Kyoto 606-8501, Japan

## Abstract

As the world's population ages, the number of patients with Alzheimer's disease (AD) is predicted to increase rapidly. The presence of neurofibrillary tangles (NFTs), composed of hyperphosphorylated tau protein, is one of the neuropathological hallmarks of AD brain. Since the presence of NFTs is well correlated with neurodegeneration and cognitive decline in AD, imaging of tau using positron emission tomography (PET) and single-photon emission computed tomography (SPECT) is useful for presymptomatic diagnosis and monitoring of the progression of AD. Therefore, novel PET/SPECT probes for the imaging of tau have been developed. More recently, several probes were tested clinically and evaluated for their utility. This paper reviews the current state of research on the development and evaluation of PET/SPECT probes for the imaging of tau in AD brain.

## 1. Introduction

Alzheimer's disease (AD) is the most common form of irreversible dementia of the elderly. The number of AD patients was estimated at 26.6 million worldwide in 2006. As the world's population ages, the prevalence of AD is predicted to grow fourfold, to 106.8 million in 2050 [1, 2]. Senile plaques (SPs), composed of *β*-amyloid (A*β*) peptides, and neurofibrillary tangles (NFTs), composed of hyperphosphorylated tau proteins, are two neuropathological hallmarks of AD brain [3, 4]. However, there is no simple and definitive diagnostic method to detect SPs and NFTs in the brain without postmortem pathological staining of brain tissue. Therefore, the quantitative evaluation of SPs and NFTs in living brain by noninvasive techniques such as positron emission tomography (PET) and single-photon emission computed tomography (SPECT) could lead to the presymptomatic detection of AD [5–7]. Much efforts have been discussed on the development of A*β* imaging probes, and some of them, such as (*E*)-4-(N-methylamino)-4′-(2-(2-(2-[^18^F]-fluoroethoxy)ethoxy)ethoxy)-stilbene ([^18^F]florbetaben) [8, 9], (*E*)-4-(2-(6-(2-(2-(2-[^18^F]-fluoroethoxy)ethoxy)ethoxy)pyridin-3-yl)vinyl)-*N*-methylaniline ([^18^F]florbetapir) [10–12], and 2-(3-[^18^F]-fluoro-4-methylaminophenyl)benzothiazol-6-ol ([^18^F]flutemetamol) [13, 14], were approved by the US Food and Drug Administration (FDA).

Although SPs are often found in the brains of both AD and healthy controls, NFTs in the hippocampus and entorhinal cortex are well correlated with neurodegeneration and cognitive decline [18, 19]. Furthermore, the formation and deposition of NFTs occur in the early stage of AD. Therefore, imaging of tau, which is the main constituent of NFTs, by PET/SPECT is useful for the presymptomatic diagnosis and monitoring of the progression of AD. 2-(1-(2-(*N*-(2-[^18^F]-Fluoroethyl)-*N*-methylamino)naphthalene-6-yl)ethylidene)malononitrile ([^18^F]FDDNP), which is the first PET probe to visualize AD pathology, has been demonstrated to be effective in the measurement of in vivo brain cortical accumulation of both SPs and NFTs in living subjects [20–22]. Since this probe has lower binding affinity and lack of selectivity for tau aggregates, novel probes that show high selectivity for tau aggregates have been strongly desired. In this review, we summarize the current situation in the development of imaging probes for tau.

## 2. Quinoline Derivatives for Imaging of Tau

**Figure 1:**
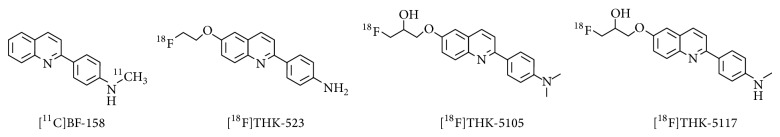
Chemical structures of quinoline derivatives for the imaging of tau [15–17].

Okamura and coworkers screened >2000 small molecules in order to develop novel probes for imaging A*β* and tau aggregates. In this search, they identified that the quinoline scaffold selectively binds to tau aggregates (Figure 1). Therefore, some compounds were tested for the in vivo imaging of tau in brain [15]. Among them, 2-[(4-methylamino) phenyl]quinoline (BF-158) showed high affinity for tau aggregates in vitro and clearly stained NFTs in the hippocampal section of AD brain. Furthermore, by in vitro autoradiography in AD brain sections, the distribution pattern of [^11^C]BF-158 correlated well with tau immunostaining in the adjacent section. The authors suggested that these results indicated the binding of [^11^C]BF-158 for NFTs in the AD brain. However, ^11^C is a positron-emitting isotope with a short *t*_1/2_ (20 min), which limits its clinical application. Therefore, they attempted to develop novel quinoline derivatives, labeled with ^18^F, having a longer *t*_1/2_ (110 min).

First, Fodero-Tavoletti and coworkers developed 6-(2-[^18^F]-fluoroethoxy)-2-(4-aminophenyl)quinolone ([^18^F]THK-523) for the quantitative imaging of tau aggregates in the human brain [16, 29]. This probe showed selective affinity for tau aggregates in vitro. By in vitro autoradiography in AD brain sections, the distribution of [^18^F]THK-523 did not conform with those of 2-(4′-[^11^C]-methylaminophenyl)-6-hydroxybenzothiazole ([^11^C]PIB) [30, 31] and 2-(2-[2-[^11^C]-dimethylaminothiazol-5-yl]ethenyl)-6-(2-[fluoro]ethoxy)benzoxazole ([^11^C]BF-227) [32], which are A*β* imaging probes. Furthermore, the distribution of [^18^F]THK-523 in AD brain section closely resembled the findings upon Gallyas silver staining and tau immunostaining. On the basis of these results, [^18^F]THK-523 was tested clinically [33]. However, the pharmacokinetics and binding characteristics of [^18^F]THK-523 might not reach the necessary optimal levels for PET probes.

**Figure 2:**
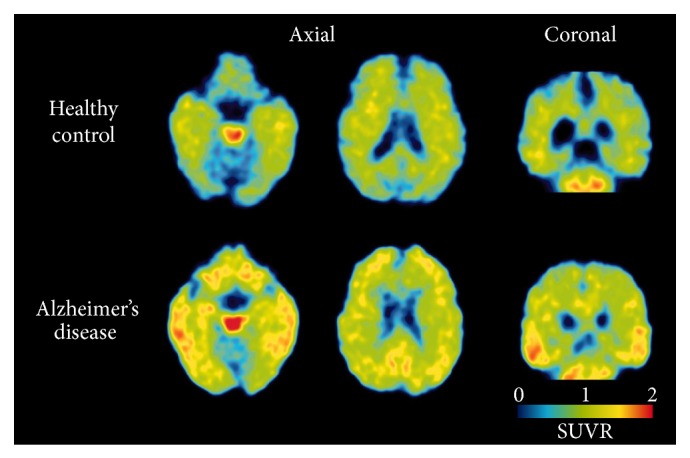
^18^F-THK5105 PET images from 60 to 80 min after injection in a healthy control subject (72 years old, CDR 0, MMSE 29) and a patient with Alzheimer's disease (68 years old, CDR 1.0, MMSE 20). Reproduced from [23] with permission, copyright 2014 Oxford University Press.

Therefore, Okamura and coworkers developed 6-[(3-[^18^F]-fluoro-2-hydroxy)propoxy]-2-(4-dimethylaminophenyl)quinoline ([^18^F]THK-5105) and 6-[(3-[^18^F]-fluoro-2-hydroxy)propoxy]-2-(4-methylaminophenyl)quinoline ([^18^F]THK-5117) for use as novel tau imaging PET probes (Figure 1) [17]. By in vitro binding assays, these compounds displayed higher affinity to tau aggregates than THK-523. By in vitro autoradiography in AD brain sections, the distributions of [^18^F]THK-5105 and [^18^F]THK-5117 coincided with Gallyas-Braak staining and tau immunostaining but not with the distribution of [^11^C]PIB and A*β* immunostaining. In the biodistribution studies in normal mice, these probes showed higher uptake to mouse brain at 2 min after injection than [^18^F]THK-523. In addition, the clearance of these derivatives from normal brain was also higher than that of [^18^F]THK-523. These results suggest that [^18^F]THK-5105 and [^18^F]THK-5117 are suitable compounds for clinical application. Therefore, first, [^18^F]THK-5105 was tested clinically and evaluated in terms of whether it could selectively bind to tau aggregates in living patients with AD [23]. The results of this study suggested that [^18^F]THK-5105 is useful for the noninvasive evaluation of tau pathology in humans (Figure 2). In addition, clinical study of [^18^F]THK-5117 is also ongoing.

## 3. Benzoimidazopyrimidine and Pyridoindole Derivatives for Imaging of Tau

**Figure 3:**
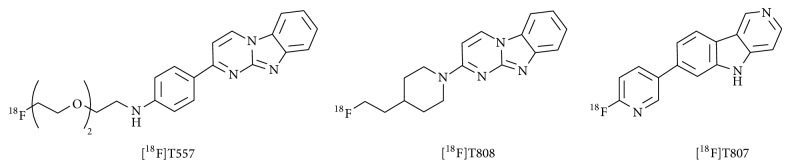
Chemical structures of benzoimidazopyrimidine and pyridoindole derivatives for the imaging of tau [24, 25].

Zhang and coworkers designed and synthesized a novel class of compounds, benzo[4,5]imidazo[1,2-a]pyrimidines, for in vivo imaging of tau in brain (Figure 3) [24]. Among numerous compounds, 4-(benzo[4,5]imidazo[1,2-*a*]pyrimidin-2-yl)-*N*-(2-(2-(2-fluoroethoxy)ethoxy)ethyl)aniline (T557) was identified as having good tau binding properties. However, imaging studies of [^18^F]T557 in rodents showed poor brain uptake. Therefore, they developed 2-(4-(2-[^18^F]-fluoroethyl)piperidin-1-yl)benzo[4,5]imidazo[1,2-*a*]pyrimidine ([^18^F]T808) as a novel imaging probe for tau. By in vitro autoradiography in AD brain sections, this probe localized in the tau-rich sections with considerable selectivity and specificity. Compared with [^18^F]T557, [^18^F]T808 rapidly crossed the blood brain barrier, followed by rapid clearance, suggesting little nonspecific binding in mice.

Xia and coworkers developed a novel class of 5H-pyrido[4,3-b]indoles and evaluated their binding affinity toward tau and their selectivity over A*β* aggregates (Figure 3) [25]. Through several rounds of lead optimization, 7-(6-fluoropyridin-3-yl)-5H-pyrido[4,3-b]indole (T807) was identified as a novel imaging probe for tau. The results of in vitro autoradiography in AD brain sections suggested that [^18^F]T807 bounded to NFTs of human AD brain with high specificity and weak or no interaction with SPs. In a biodistribution study in normal mice, [^18^F]T807 showed high brain uptake and rapid clearance from brain. In addition, [^18^F]T807 was metabolically stable in mice and [^18^F]T808 underwent slow yet acceptable de[^18^F]-fluorination. On the basis of these results, [^18^F]T807 and [^18^F]T808 were tested clinically. These preliminary results suggested that [^18^F]T807 and [^18^F]T808 have promise for the in vivo imaging of tau in AD patients [34, 35].

## 4. Benzothiazole Derivatives for Imaging of Tau

**Figure 4:**
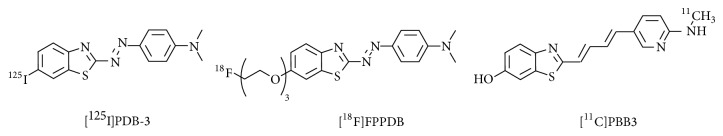
Chemical structures of benzothiazole derivatives for the imaging of tau [26–28].

Recently, Honson and coworkers screened to determine the feasibility of distinguishing tau aggregates from A*β* aggregates with a library containing 72,455 compounds [36]. They found that a phenyldiazenyl benzothiazole (PDB) derivative showed high binding affinity for tau aggregates with twofold selectivity over A*β* aggregates. Based on these findings, we designed novel radioiodinated PDB derivatives and evaluated their utility for the imaging of tau in AD brain (Figure 4) [26]. Among them, (*E*)-4-((6-iodobenzo[*d*]thiazol-2-yl)diazenyl)-*N*,*N*-dimethylaniline (PDB-3) showed high and selective binding affinity for tau aggregates in vitro. However, [^125^I]PDB-3 showed poor pharmacokinetics in normal mouse brain. Therefore, we developed (*E*)-4-((6-(2-(2-(2-[^18^F]-fluoroethoxy)ethoxy)ethoxy)benzo[*d*]thiazol-2-yl)diazenyl)-*N*,*N*-dimethylaniline ([^18^F]FPPDB), which is a novel probe for the imaging of tau, to improve the in vivo pharmacokinetics of PDB-3 (Figure 4) [27]. [^18^F]FPPDB showed higher initial brain uptake and faster clearance from brain than [^125^I]PDB-3. However, compared with PDB-3, FPPDB displayed less selectivity between tau and A*β* aggregates. In addition, by in vitro autoradiography in human AD brain sections, the distribution of [^18^F]FPPDB corresponded with that of in vitro immunohistochemical staining against phosphorylated tau, but it also matched to immunohistochemical staining against A*β*. Therefore, further structural optimization based on PDB scaffold is necessary to develop more useful probes for the in vivo imaging of tau in AD brains.

**Figure 5:**
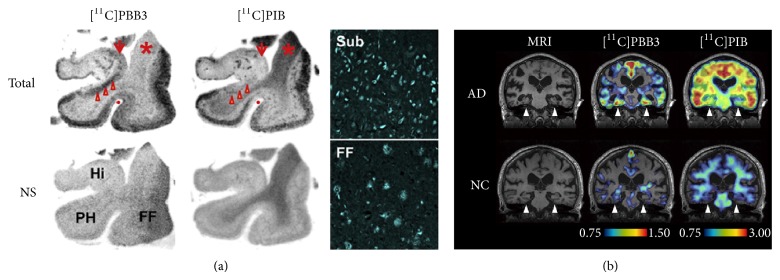
Accumulation of [^11^C]PBB3 in the hippocampal formation of AD patients revealed by in vitro autoradiography (a) and in vivo PET (b). Reproduced from [28] with permission, copyright 2014 Elsevier.

More recently, Maruyama and coworkers developed novel tau imaging probes, phenyl/pyridinyl-butadienyl-benzothiazole/benzothiazolium derivatives, for the imaging of tau in AD brain (Figure 4) [28]. Among them, 2-((1*E*,3*E*)-4-(6-(methylamino)pyridin-3-yl)buta-1,3-dienyl)benzo[*d*]thiazol-6-ol (PBB3) clearly stained tau aggregates in tau model mouse brain sections and human AD brain sections. By ex vivo autoradiography, PBB3 selectively labeled the brain stem and spinal cord of tau model mice harboring neuronal tau inclusions. These accumulations could be detected with a micro-PET system in vivo. In addition, by in vitro autoradiography in AD hippocampus, a notable difference in the distribution between [^11^C]PBB3 and [^11^C]PIB was observed in the tau-rich sections (Figure 5(a)). Since these results suggested that [^11^C]PBB3 is a useful PET probe for the imaging of tau, they conducted an exploratory clinical PET study for patients with probable AD and healthy controls (Figure 5(b)). The result of this study supported the potential utility of [^11^C]PBB3 for clarifying correlations between the distribution of tau deposition and the symptomatic progression of AD.

## 5. Other Probes for Imaging of Tau

Rojo and coworkers prepared two benzimidazole derivatives, astemizole and lansoprazole, to evaluate their interactions with tau aggregates [37]. Since these compounds showed high and specific affinity with tau aggregates, radiolabeled astemizole [38] and lansoprazole [39, 40] derivatives were reported. In particular, [^18^F]*N*-methyl lansoprazole is the lead compound for progression into clinical trials because this probe showed rapid brain uptake in nonhuman primates, favorable kinetics, low white matter binding, and selectivity for binding to tau aggregates over A*β* aggregates. In addition, some compounds were also developed as novel PET and SPECT probes for the imaging of tau [41–44].

## 6. Conclusion

In the past decade, novel PET and SPECT probes for the imaging of tau were developed and evaluated for their utility. Among them, several compounds including [^18^F]THK-523, [^18^F]THK-5105, [^18^F]T807, [^18^F]T808, and [^11^C]PBB3 were tested clinically. The results showed their feasibility for imaging tau aggregates for the diagnosis of AD. Therefore, we hope that further studies of these probes should lead to novel methods for presymptomatic diagnosis and monitoring of the progression of AD.
